# Current Understanding of Osteoimmunology in Certain Osteoimmune Diseases

**DOI:** 10.3389/fcell.2021.698068

**Published:** 2021-08-17

**Authors:** Anqi Zhou, Bingfeng Wu, Hui Yu, Yufei Tang, Jiayi Liu, Yinan Jia, Xiaoyu Yang, Lin Xiang

**Affiliations:** ^1^State Key Laboratory of Oral Diseases and National Clinical Research Center for Oral Diseases, West China Hospital of Stomatology, Sichuan University, Chengdu, China; ^2^Department of Oral Implantology, West China Hospital of Stomatology, Sichuan University, Chengdu, China; ^3^Department of Orthodontics, West China Hospital of Stomatology, Sichuan University, Chengdu, China

**Keywords:** osteoimmunology, RANKL/RANK/OPG, rheumatoid arthritis, periodontitis, bone repair, dental implantation

## Abstract

The skeletal system and immune system seem to be two independent systems. However, there in fact are extensive and multiple crosstalk between them. The concept of osteoimmunology was created to describe those interdisciplinary events, but it has been constantly updated over time. In this review, we summarize the interactions between the skeletal and immune systems in the co-development of the two systems and the progress of certain typical bone abnormalities and bone regeneration on the cellular and molecular levels according to the mainstream novel study. At the end of the review, we also highlighted the possibility of extending the research scope of osteoimmunology to other systemic diseases. In conclusion, we propose that osteoimmunology is a promising perspective to uncover the mechanism of related diseases; meanwhile, a study from the point of view of osteoimmunology may also provide innovative ideas and resolutions to achieve the balance of internal homeostasis.

## Introduction

The skeletal system and immune system seem to be independent but are in fact inseparable and closely related. On the one hand, as the primary hemopoietic organ, bone marrow nourishes and provides a unique environment for the growth of hematopoietic stem cells (HSCs), the common origin of all types of immune cells (Walsh et al., 2018), which establishes the structural basis of regulation on the immune system by bone. On the other hand, the activity of immune cells is of great significance for the occurrence and the development of pathological bone damage diseases, such as rheumatoid arthritis (RA), periodontitis, delayed bone regeneration, and bone abnormalities in other infectious diseases. Therefore, cells from the immune and skeletal systems share the same microenvironment and interact extensively. Arron and Choi (2000) established the concept of osteoimmunology first to describe the interaction between the skeletal and immune systems, while the specific definition of osteoimmunology was not clarified. Takayanagi (2007) defined osteoimmunology and highlighted its interdisciplinarity, and the scope of osteoimmunology was extended into the interaction between a wide range of cells from the skeletal and immune systems. In recent years, with the advancements in studies on the crosstalk between the two systems and our cognition, the concept of osteoimmunology has been further updated. Okamoto et al. (2017) proposed that osteoimmunology emphasized the interaction between the two systems from the macro level to the micro level as well as the inner mechanism in both physiologic and pathologic conditions, and it involved the research progress on therapeutic strategies for relevant diseases, which is a relatively comprehensive summary of osteoimmunology so far. Since osteoimmunology builds the bridge between the skeletal and immune systems, it is of great value to discuss the regulatory role that osteoimmunology plays in a series of essential biological processes, suggesting promising perspectives for studies and potential therapies related to skeletal and immune system diseases.

The skeletal system and immune system share various cytokines and signaling pathways. Scientists have uncovered the significant role of the receptor activator of nuclear factor kappa B ligand (RANK ligand or RANKL)/RANK/osteoprotegerin (OPG) axis in osteoimmunomodulation in the past few years (Okamoto et al., 2017; Tsukasaki and Takayanagi, 2019). Besides, there are an increasing number of novel studies aiming to reveal more promising key cytokines and signaling pathways that connect the skeletal and immune systems and are involved in the research scope of osteoimmunology, such as tumor necrosis factor-α (TNF-α) (Bertolini et al., 1986), interferon-γ (IFN-γ) (Smith et al., 1987), interleukin-1 (IL-1) (Gowen et al., 1983), IL-4 (Shioi et al., 1991), IL-6 (Löwik et al., 1989), IL-17 (Kotake et al., 1999), IL-20 (Hsu et al., 2011), bone morphogenetic protein (BMP) (Lories et al., 2003), sphingosine-1-phosphate (S1P)-S1P receptor-1 (S1PR1) signaling (Xiao et al., 2019), Notch signaling (Liotta et al., 2008), mitogen-activated protein kinase (MAPK) pathway (Chae et al., 2001), dependent or independent on RANKL/RANK/OPG signaling pathway, which further enriches our understanding of osteoimmunology.

In this review, we will summarize the many interactions between cells of the skeletal and immune systems during the development of certain typical osteoimmune diseases as well as the molecular mechanism behind them according to the mainstream advancing studies. We would like to stress the importance of discussing these biological processes from the perspective of osteoimmunology in future research, so as to achieve the balance of internal homeostasis in the skeletal and immune systems.

## Co-Development of the Skeletal and Immune Systems

### Shared Molecular Mechanism Between Osteoimmune Cells in Osteoclastogenesis

**FIGURE 1:**
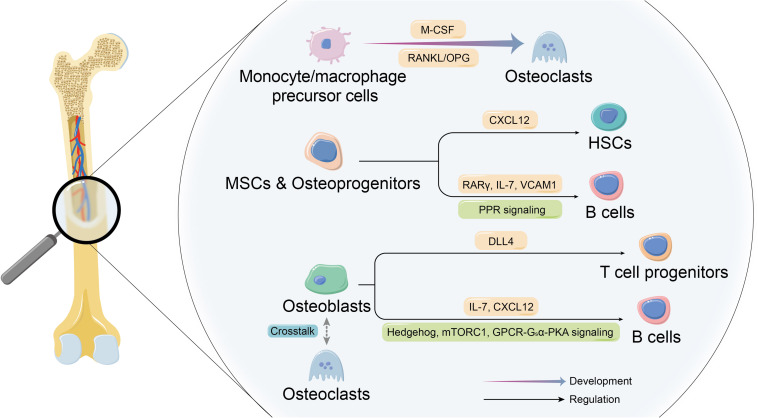
Co-development of skeletal and immune systems. The co-development of the skeletal system cells and immune system cells constitutes the basis of theory of osteoimmunology. On the one hand, certain immune cells are the essential origin of osteoclasts. On the other hand, skeletal system cells make up the unique niches in bone marrow to participate in forming the indispensable microenvironment for immune cells and affect the their development and biological behaviors.

Osteoclasts are the dominant bone resorptive cells in bone metabolism (Figure 1). The dynamic balance of bone formation and resorption driven respectively by the activity of osteogenic cells and osteoclasts is the key to physiologic bone remodeling and bone homeostasis. Under the physiological conditions, osteoclasts are multinucleated giant cells formed by the fusion of monocyte/macrophage precursor cells derived from myeloid progenitor cells in bone marrow with the indispensable involvement of macrophage colony-stimulating factor (M-CSF) and RANKL (Kong et al., 1999; Suda et al., 1999). Immature dendritic cells (DCs) are also recognized as the precursor of osteoclasts besides monocyte/macrophage precursor cells (Rivollier et al., 2004; Gallois et al., 2010), while researchers have revealed that the hypotheses of monocyte-origin and DC-origin are probably not mutually exclusive. Tsukasaki et al. (2020) discovered the transient expression of CD11c (the marker of DCs) during the process of osteoclastogenesis by the scRNA-seq analysis, and the specific deletion of RANK in CD11c^+^ cells led to inhibition of osteoclastogenesis, which strongly suggested that DC-like precursors were the indispensable transitional state between monocytic precursors and the mature osteoclasts. Furthermore, in the past few years, accumulating evidence showed that certain mesenchymal cells also serve as RANKL-producer cells that are required for bone metabolism and the progress of osteoimmune diseases. At the beginning, osteoblast is recognized as the cellular source of RANKL that plays an essential role in osteoclastogenesis (Yasuda et al., 1998). The dynamic tracking of RANKL *in vitro* has revealed that RANKL mRNA and protein are localized in osteoblasts and immature osteocytes, and the expression of RANKL decreases with the maturation of osteoblasts (Kartsogiannis et al., 1999), while some studies have indicated the opposite results that the main producers of RANKL in bone remodeling are hypertrophic chondrocytes and osteocytes rather than osteoblasts or their progenitors, which do not contribute to osteoclastogenesis (Xiong et al., 2011). A more specific description on the localization of cellular source of RANKL during the development of osteoclasts may need further clarification. Besides, the latest evidence demonstrated another committed cell population from adipogenic lineage that regulates bone resorption via RANKL. Yu et al. identified novel marrow adipogenic lineage precursors (MALPs), making up a ubiquitously distributed 3D network in bone marrow to maintain bone marrow capillary structure and suppress osteogenesis. The recent published paper further revealed that MALPs expressed RANKL and showed spatial specificity in controlling the osteoclastogenesis during bone remodeling, confirming the role of MALPs in bone resorption in physiologic and pathologic states (Yu et al., 2021). Additionally, a unique subset of macrophages named as arthritis-associated osteoclastogenic macrophages (AtoMs) was identified, which functioned as the precursor of osteoclasts in arthritis (Hasegawa et al., 2019). What is more, it is worth noticing that monocyte/macrophage precursor cells from different origins are tissue-specific. Recently, scientists have revealed that yolk-sac macrophages of erythromyeloid progenitor (EPM) origin produced neonatal osteoclasts that are independent of HSC lineage (Gomez Perdiguero et al., 2015; Yahara et al., 2020), providing a further and more precise explanation of the developmental mechanism of osteoclasts.

Moreover, over the last few years, researchers have been paying more attention to the function of cells involved in osteoimmunology as well as the underlying molecular mechanism and associating it with dysregulated bone homeostasis and bone diseases (Yang and Wan, 2019). M1/M2 dichotomy is well known as the classic classification pattern of macrophage that immature macrophages are subdivided into pro-inflammatory M1 and anti-inflammatory/regulatory M2 according to the activator, surface maker, secretion, and function, playing their distinct roles in regulating osteoimmune microenvironment (Yamaguchi et al., 2016; Brown et al., 2017). Besides, the diversity between the two subtypes also includes the osteoclastogenic property. Yang et al. (2019) observed the osteoclastogenic potential of the two subtypes, and the results suggested that M2 macrophages were more efficient osteoclast precursors than M1 macrophages, which was attributed to a higher level of interferon regulatory factor 5 (IRF5) expression. Similarly, in the pathological condition, with the decreased level of estrogen and elevated level of RANKL, M2 macrophages are stimulated to differentiate to osteoclasts in ovariectomized (OVX) mice, while M1 macrophages are not influenced, hence the improved M1/M2 ratio, which can be rescued by estrogen or estrogen receptor α (Erα)-selective agonist (Dou et al., 2018). In addition to the discrepancy of osteoclastogenic potential, the direct regulatory role of the two subtypes on osteoclast precursors cannot be ignored. M1 macrophages were discovered to inhibit osteoclastogenesis of RAW264.7 cells and bone marrow cells via secreting IFN-γ and IL-12, while M0 and M2 were not, contributing to the complexity of osteoclastogenesis mechanism in settings of osteoimmune diseases in which infiltration of macrophages is involved (Yamaguchi et al., 2016).

### Osteoblast Lineage Provides Indispensable Unique Niches for Immune Cells

Bone marrow offers the specific microenvironment where immune cells differentiate, which is known as “niches.” These niches mainly consist of heterogeneous stromal cells, many of which are of mesenchymal origin, including various subsets of osteoblast lineage (Panaroni and Wu, 2013), establishing the histological basis of osteoblast lineage playing a supportive and collaborative role in the development, maintenance, and mobilization of immune cells and their precursors. For a long time, scientists have been committed to the study on the characteristics of immune cells in the special bone marrow niches, while the growth mechanism of immune cells and the specific regulatory function of niches are still not fully clarified.

Osteoblast lineage cells of different maturity stages play different roles, and the precise regulation at different stages of osteoblastogenesis is required for optimal development procedure of immune cells (Wu et al., 2008). According to an article published on *Nature*, after selectively depleting Nestin^+^ mesenchymal stem cells (MSCs) in mice, Méndez-Ferrer et al. (2010) observed a ∼4-fold reduction in the activity of bone marrow HSCs. In the absence of Nestin^+^ MSCs, the homing of HSCs to bone marrow was markedly reduced, which might be related to the considerably high level of key gene expression that regulates HSC behaviors in MSCs. It is further demonstrated that CXC-motif chemokine ligand 12 (CXCL12) in early mesenchymal progenitors is essential for the maintenance of HSCs (Sugiyama et al., 2006), and B lymphopoiesis is regulated by MSCs retinoic acid receptor γ (RARγ) activity (Green et al., 2018). Moreover, parathyroid hormone (PTH)/PTH-related peptide receptor (PPR) signaling in osteoprogenitors plays an essential role for B lymphopoiesis by expressing IL-7 and the mobilization of B cells by expressing vascular cell adhesion molecule 1 (VCAM1) (Panaroni et al., 2015).

The effects of osteoblasts on making up the unique microenvironment for immune cells seem to be understood more thoroughly. Scientists have proved that regulation of B lymphopoiesis is mediated by Hedgehog (Hh) signaling, mechanistic target of rapamycin complex 1 (mTORC1) signaling, and G protein-coupled receptors (GPCR)-G_*s*_α-protein kinase A (PKA) pathway in osteoblasts, thus influencing the expression of key cytokines that support the development of B cells, such as IL-7 or CXCL12 (Wu et al., 2008; Lu et al., 2018; Martin et al., 2018; Wang et al., 2018). Moreover, the generation of T-cell progenitors has been demonstrated to be promoted by osteoblasts-derived Notch ligand delta-like 4 (DLL4).

It is also worth noticing that osteoclast is an important component of the niche for immune cells. It has been shown that osteoclasts contribute to mobilization of HSCs by influencing endosteal niche components (Kollet et al., 2006), and the role of osteoclasts as niches for immune cells can also be suggested indirectly by B lymphocytopenia in osteopetrosis (Mansour et al., 2011). However, there are some other studies that show opposite conclusion. For example, osteopetrotic mice present normal mobilization of HSCs in response to the stimulation of granulocyte CSF (G-CSF) (Miyamoto et al., 2011), suggesting the regulatory effects of osteoclasts on immune cells in bone marrow may be applicable in certain conditions. Furthermore, rather than regulating the fate of immune cells directly, osteoclasts contribute to supporting the development of immune cells by influencing biologic behaviors of osteoblasts, thus changing the bone marrow microenvironment (Mansour et al., 2011, 2012). In a word, the skeletal system provides indispensable unique niches in bone marrow for growth of immune cells through a variety of direct and indirect ways, and the study on osteoimmune niche cells interaction is promising to reveal the inner mechanism of immune cells development, so as to achieve the balance of bone marrow internal homeostasis and provide innovative ideas and resolutions for correlated diseases.

## Osteoimmunology in Typical Bone Abnormalities and Bone Regeneration

### Rheumatoid Arthritis

**FIGURE 2:**
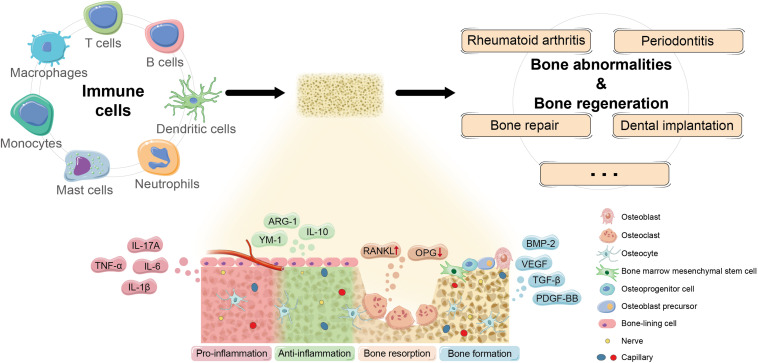
Potential mechanism of osteoimmunomodulation in bone abnormalities and bone regeneration. Immune cells regulate the occurrence and development of bone abnormalities and bone regeneration by impacting the osteoimmune microenvironment through a series of key molecules and pathways to influence the biological behaviors of skeletal system cells. Currently, more and more promising cytokines and signaling that participate in osteoimmunomodulation have been discovered, such as pro-inflammatory TNF-α, IL-1β, IL-6, and IL-17A; anti-inflammatory IL-10, ARG-1, and YM-1; osteoclastogenic RANKL/OPG; and bone repair signals like TGF-β, BMP-2, PDGF-BB, and VEGF.

The interdisciplinary study on RA seems to be the most advanced among osteoimmune diseases (Figure 2). The main pathological features of RA include proliferation of synovial lining cells, infiltration of an abnormally large number of inflammatory cells, advanced angiogenesis, pannus formation, and destruction of cartilage and bone tissue.

As for the cellular and molecular mechanisms, the activation of T lymphocytes induced by antigen-presenting cells (APCs) is the initial and key step to stimulating bone resorption in the inflamed synovium. T helper 17 (T_H_17) cell that is defined by characteristic expression of IL-17 is recognized as a specialized subset that induces osteoclastogenesis at the interface of bone and activated immune system after examining a variety of T_H_ cells (Sato et al., 2006). Induced by elevated IL-17, various innate immune cells like macrophages, mast cells, and neutrophils further impact on RANKL/OPG ratio and then the development of osteoclasts by secreting effective osteoclastic cytokines such as TNF-α, IL-1, and IL-6 (Roser-Page et al., 2018; Tsukasaki and Takayanagi, 2019). Moreover, as a group of lymphocytes that negatively regulates immune response, regulatory T (Treg) cells usually play an important role in restraining excessive immune response and maintaining microenvironment homeostasis, which has been verified in Treg cells from human tissue (Delacher et al., 2021). Treg cells are the crucial component in T cells that mediated anti-osteoclastogenesis in RA, and ameliorating the imbalance of Treg/T_H_17 has become a popular therapeutic target in forefront studies (Geng et al., 2017; Jin et al., 2018; Shi et al., 2020; Shang et al., 2021). Moreover, Forkhead box protein P3 (FOXP3) is considered as a specific transcription factor that plays a crucial role in the development and the function of Treg cells by regulating different genes directly, and researchers have proposed its relations to the pathogenesis of RA. Nie et al. (2013) discovered that TNF-α stimulated the transcriptional and enzymatic activity of protein phosphatase 1 (PP1) in the inflamed synovium, leading to dephosphorylation at the specific site in FOXP3, resulting in the impaired protective function of Treg cells and the imbalance between T_H_17 and Treg cells. Meanwhile, Komatsu et al. (2014) revealed that the instability of FOXP3 expression was also attributed to excessive IL-6 produced by synovial fibroblasts, another essential cell population involved in the basic osteoimmunological progress of RA. The mechanism of synovial fibroblasts contributing to immune cells-mediated osteoclastogenesis in RA includes not only their weakened capacity to inhibit the activity of T_H_ cells but also the stimulated expression of inflammatory factors by IL-17 (Hirota et al., 2018; Kaul et al., 2020), which form a vicious circle in the osteoclastogenesis and aggravate bone damage of individuals with RA. In particular, it has been demonstrated that synovial fibroblasts serve as the main source of RANKL for bone destruction in RA instead of T cells (Danks et al., 2016). Additionally, with the development of genomic medicine, scRNA-seq has become a popular method to identify the characteristics and biomarkers of complex disease such as RA. Based on the recent scRNA-seq analyses of inflamed synovium samples of RA mouse models and patients, sublining fibroblasts are considered as a major source of pro-inflammatory factors like IL-6, and the expansion of a distinct subset of PDPN^+^ CD34^–^THY1^+^ sublining fibroblasts may be pathogenic in RA tissues, suggesting the potential therapeutic target of RA (Mizoguchi et al., 2018; Gawel et al., 2019; Zhang et al., 2019). The latest evidence also indicates that the position identity of synovial fibroblasts is regulated by Notch signal from endothelial cells (Wei et al., 2020).

Scientists have uncovered the molecular mechanisms of autoimmune response in RA relatively clearly, while the potential therapeutic approach of damaged bone needs further discovery. Culemann et al. (2019) identified a group of CX_3_C motif chemokine receptor 1-positive (CX_3_CR1^+^) tissue-resident macrophages in synovial lining and proposed that they could form a dynamic membrane-like structure by tightly connecting with each other, establishing a protective barrier to inhibit the inflammatory reaction. Such results uncovered the multiple functions of macrophage in addition to its pro-inflammatory effect in RA, as well as suggested the promising therapeutic value of macrophage. As mentioned before, TNF-α induces impaired function of Treg cells via FOXP3 and inhibits their protective role in the synovium, while TNF receptor type II (TNFRII), the receptor type that mediated the anti-inflammatory effects of TNF, has been demonstrated to participate in maintaining the stable expression of FOXP3, suggesting that the potential mechanism of anti-TNF agent in RA treatment may be attributed to the expansion of TNFRII^+^ Treg cells (Santinon et al., 2020). Moreover, because oxidative stress and RA are closely related pathologically, antioxidant therapy has also been considered as a prospective strategy of RA (Khojah et al., 2016; Jabbari et al., 2020). In addition, the Hippo-Yes-associated protein (YAP) signaling pathway regulates the expression of target genes to affect a series of physiological activities through responding to other intracellular and extracellular signals, consequently playing important roles in cell proliferation, differentiation, and apoptosis, which suggests its indispensable impacts on osteogenesis and immune regulation (Liu et al., 2016; Xiang et al., 2018; Wang et al., 2019). Therefore, it is remarkable that there is extensive crosstalk between Hippo signaling and oxidative stress signaling, and a positive feedback mechanism between YAP and reactive oxygen species (ROS) in oxidative stress disease has been proven (Li et al., 2019; Zheng et al., 2020). Recent researches have shown that the suppression of YAP, the main effector of Hippo pathway, leads to inhibiting the pathogenic behavior of fibroblast synovial cells via reducing their migration and invasion, which suggests Hippo-YAP signaling as a prospective therapeutic target of RA (Bottini et al., 2019; Zhou W. et al., 2020). Further study is encouraged to concentrate on the crosstalk between Hippo-YAP and oxidative stress in osteoimmune regulation to RA, as well as the prospective value of antioxidant therapy it mediates.

### Periodontitis

Apart from periodontitis, there are few conditions when infection takes place around the bone, which makes periodontitis a unique disease in osteoimmunology (Tsukasaki, 2020). Unlike autoantigen-induced RA, periodontitis starts with polymicrobial synergy and dysbiosis (Kuboniwa et al., 2017), which trigger innate immune response via bacteria and their components like lipopolysaccharide (LPS). LPS is the activator of M1 macrophage, which, as mentioned before, is the pro-inflammatory subtype, secreting pro-inflammatory cytokines like TNF-α and IL-1β, thus stimulating inflammation chronic protraction. More than macrophages, DCs and T lymphocytes are also involved in the development of periodontitis. A cross-sectional study suggests that enhanced expression of IL-6 contributed to promoted number of immature DCs infiltrating in patients’ periodontal tissue and in turn act on inflammatory response, though with the development of the disease, the number of these cells would gradually decrease (Souto et al., 2014). Moreover, clinical trials also show the increased number of peripheral and local T_H_1 and T_H_17 lymphocytes, the essential T_H_ cell populations in nuclear factor kappa B (NF-κB)-dependent pathogenesis of periodontitis, as well as promoted the expression level of T-bet, IFN-γ, and IL-17A in chronic periodontitis (Chen et al., 2016; Monasterio et al., 2019). Specifically, like macrophages, under the stimulation from bacteria and its proteins, DCs also induce T_H_1 or T_H_17 lymphocytes to upregulate the level of TNF-α and IL-1β, while they activate Th2 and Treg cells to play a protective role against inflammation and bone destruction simultaneously in periodontal microenvironment (Song et al., 2018). Activated by IL-1β, IL-6, and IL-23, T_H_17 cells produce IL-17 family cytokines including IL-17A, which is demonstrated to be the primary mediator of immunopathology in the context of periodontitis and periodontitis-related systemic diseases (Moutsopoulos et al., 2012; Dutzan et al., 2018; Samuel et al., 2019), while IL-17E, another subtype of IL-17, may play an opposite role in the occurrence and development of periodontitis (Awang et al., 2014). In addition, a Tregs–T_H_17 conversion occurs during inflammation of periodontitis, and the reduction of the conversion has been considered as an effective target for periodontitis therapy (Rajendran et al., 2019). Stimulated macrophages, DCs, and T cells promote the expression level of RANKL in periodontal ligament cells and osteoblasts through their corresponding pro-inflammatory cytokines so as to initiate bone resorption performed by osteoclasts, with OPG being degraded as well (Tsukasaki, 2020), resulting in reduced RANKL/OPG ratio, which leads to alveolar bone lesion even tooth loss. Nowadays, there are more and more advanced researches focusing on the potential key molecule or signaling pathway targeted at regulating osteoimmune microenvironment in periodontitis (Goes et al., 2019; Li et al., 2020; Wang L. et al., 2020), which may provide novel perspectives and contribute to the therapeutic approaches for periodontitis based on osteoimmunomodulation. For instance, in a recent study, Wu et al. (2020) revealed the new AKT2/c-Jun N-terminal kinase (JNK)1/2/c-jun and AKT2/miR-155-5p/DET1/c-Jun signaling pathways that played a role in altering macrophage polarization and regulating periodontitis inflammation and AKT2 promoted M2 polarization of macrophages, suggesting a novel strategy for periodontitis treatment. As mentioned before, T_H_1 and T_H_17 are both vital in aggravating periodontitis, while the T_H_17 pathway has been found to be more predominant in apical periodontitis in diabetic rats than T_H_1 pathway, suggesting a more precise target in pathological mechanism of individuals with both apical periodontitis and diabetes (Samuel et al., 2019).

### Bone Repair

Immune modulation has been considered as a therapeutic strategy in bone regeneration for a long time. The process of bone repair follows a certain procedure that reduces to activation, resorption, and formation with the involvement of a variety of bone and immune cells in coordination.

In recent years, more and more attention has been paid to the role of macrophages in bone remodeling and targeting osteogenic cell–macrophage interaction, which have been proved as effective strategies to regulate the fate of stem and progenitor cells so as to facilitate bone repair. Besides, the cellular interaction and signal crosstalk are reflected not only between macrophages and osteogenic cells but also between macrophages and angiogenic cells, with the latter playing a regulatory role in bone metabolism in an indirect manner (Dohle et al., 2014). Macrophages recruit tissue repair cells such as MSCs and fibroblasts by secreting key cytokines and chemokines and ultimately accelerating the procedure of bone regeneration surrounding the bone defect. It has been found that macrophages polarize along a transient polarization spectrum to adapt and react to distinct microenvironment (Brown et al., 2017). As mentioned before, classically activated macrophages and alternatively activated macrophages are the two mainstream subtypes of macrophages commonly known as M1 and M2, which are marked by CD86 and CD206/CD163, respectively (Goerdt and Orfanos, 1999; Mantovani et al., 2002; Mosser and Edwards, 2008). Stimulated by LPS and IFN-γ, M1 macrophages secrete pro-inflammatory factors like TNF-α, IL-1β, IL-6, IL-8, and inducible nitric oxide synthase (iNOS) to aggravate the process of inflammation and lead to tissue destruction. On the contrary, M2 is induced by IL-4 and IL-13, participating in inhibiting inflammation progress and promoting angiogenesis and tissue remodeling via secreting anti-inflammatory factors such as IL-10, ARG-1, and YM-1 as well as tissue repair signals like transforming growth factor-β (TGF-β), BMP-2, platelet-derived growth factor-BB (PDGF-BB), and vascular endothelial growth factor (VEGF) (Zhou A. et al., 2020). Although there are more and more subtypes of macrophages and other categories that have been proposed (Mantovani et al., 2004; Murray et al., 2014; Chávez-Galán et al., 2015), the classic M1/M2 dichotomy is still popular and practical in recent work (Wang C. W. et al., 2020; Wu et al., 2020), which is likely attributed to the certain hallmarks of M1 and M2 phenotype that can be observed. Moreover, there is some evidence showing that M1/M2-related genes participate in the dynamic procedure of bone regeneration on bone–implant interface (Yuan et al., 2020). Therefore, it is of great value to further clarify the mechanism of macrophage-mediated bone metabolism and conceptualize the promotion of M1 to M2 transformation as a potential method of bone repair.

Another strategy of studying monocyte/macrophage-lineage cells mediated bone repair nowadays is focusing on a specific subpopulation. Tartrate-resistant acid phosphatase-positive (TRAP^+^) cells represent bone resorbing cells in bone metabolism and are of particular interest to scientists for a long time. Several recent studies have revealed the multiple functions of these cells. Gao et al. (2019) proved that macrophage-lineage TRAP^+^ cells secrete PDGF-BB to recruit periosteum-derived cells (PDCs) and upregulate the expression of periostin, promoting periosteal osteogenesis and maintaining periosteum homeostasis. Moreover, Vi et al. (2018) identified differential proteins secreted by young and aged macrophages, among which low-density lipoprotein receptor-related protein 1 (Lrp1) was produced by young macrophages and played a positive role in coordinating bone fracture healing, suggesting a novel strategy for rejuvenation of bone repair.

More than macrophages, T cells of adaptive immune response also actively participate in the process of bone repair. In the early stage of inflammation, T lymphocytes are rapidly recruited to the injured site, among which the cytotoxic T lymphocytes are relatively abundant (Könnecke et al., 2014; Tätting et al., 2017). The coordinated inflammatory reaction is an inevitable procedure in regular process of bone repair. The initial infiltration of T cells will gradually disappear, and the second wave gathers in the callus about 2 weeks after bone fracture to play a repairing role and support bone regeneration (Könnecke et al., 2014). Different populations of T cells have different impact, and T_H_1 and T_H_17 are usually regarded as pro-inflammatory subtypes and T_H_2 and Treg cells are anti-inflammatory ones (Tian et al., 2021). Although the understanding of the paradigm has been updated and enriched now, it is well acknowledged that the abnormal transformation of T-cell subsets leads to prolonged inflammatory reaction and unsatisfactory repairing outcome, and their balance is particularly important for favorable bone regeneration (Schlundt et al., 2019). Additionally, γδ T cells were discovered to contribute to bone healing in recent years. After bone injury, the number of γδ T cells grows rapidly, and they secrete IL-17A to promote osteogenic differentiation (Ono et al., 2016), which may be stimulated by IL-1β, IL-23, complement C5a, etc. (Sutton et al., 2009; Han et al., 2011), while there are still many mysteries of the molecular mechanism that needs further exploration.

On the one hand, the findings above suggest that the mechanism of monocyte/macrophage lineage cells and T cells in regulating bone regeneration deserves further clarification. On the other hand, more attention could be paid to the interaction between innate and adaptive immune systems that are involved in the procedure of bone repair as well.

### Dental Implantation

In fact, the osseointegration of dental implants is essentially the process of bone repair that occurs on bone–implant interface and follows similar biological procedures, which osteogenesis, angiogenesis, and immune response-related cells and bioactive factors are involved in, synergizing the procedure of peri-implant bone repair. The immune response driven by infiltrated cells surrounding the implants is critical for early and long-term stabilization of osseointegration. Dynamic tracking of peri-implant region has revealed that as soon as the implantation occurs, coagulation response is induced and innate immune response follows during the first 4 weeks, and then bone remodeling is initiated (Vanegas-Acosta et al., 2010; Trindade et al., 2018). In peri-implant microenvironment, lack of immune response leads to residual cellular debris after implantation, which impairs osseointegration; on the contrary, the continuous activation of the immune system results in delayed bone regeneration and even osseointegration failure (Williams, 2008; Klopfleisch, 2016). Therefore, regulating the microenvironment is the crucial point, and it is necessary to closely follow the immune response of peri-implant immune cells to the changes in the local microenvironment.

However, what is special about the study on osteoimmunology-mediated implant osseointegration compared with regular bone repair is that osseointegration includes foreign-body reaction (FBR) to titanium implants, so we cannot ignore the inevitable impact of artificial biomaterial to mineral homeostasis of the jaw. Trindade et al. (2018) observed from the histologic and molecular levels and discovered that M2 macrophages were induced with initial osteogenesis at 10 days after the implantation occurred; and the innate immune system including the complement system, neutrophils, M1 and M2 macrophages, and group 2 innate lymphoid cells were triggered at 4 weeks with more mature and organized cortical bone formation. Until now, dentists still face the dilemma of undesirable biocompatibility and commit to designing a clinical-friendly implant surface and exploring effective methods to blend bioactive factors in dental implants. In recent years, the osteoimmunomodulation has been viewed as an available strategy in the implant modification instead of enhancing osteogenesis directly (Gao et al., 2020; Zhao et al., 2021; Zhu et al., 2021), and we propose that in future study, researchers can further explore the synergistic effect of osteogenesis, angiogenesis, immune response, and even neurogenesis to accelerate the osseointegration and promote the stability of dental implants. Besides FBR, peri-implant mucositis and peri-implantitis caused by bacteria invasion also draw clinicians’ attention for their potential impacts on stable osseointegration, while recent studies questioned if the cause–effect relationship really existed between plaque accumulation and the occurrence of peri-implant inflammation and between peri-implant inflammation and marginal bone loss (Albrektsson et al., 2019; Coli and Jemt, 2021). These ambiguous issues need further clarification by clinical trials and long-term follow-up.

Moreover, researchers should consider the physiological particularity of the jaw. First of all, the maxilla develops through intramembranous ossification, and the mandible grows through intramembranous and endochondral ossification (Jiang et al., 2014), whereas the growth of long bone such as the femur, another commonly used model in the study on implant osseointegration besides the jaw, accords with endochondral ossification. On the other hand, the osteogenic property of intramembranous ossification may be influenced by developmental origins. Ichikawa et al. (2015) found the superior bone regenerative capacity of the periosteum of the jaw compared with periosteum derived from other sites, presumably since the jaw maintains some properties of neural crest that contribute to better bone repair outcomes. In a word, the discrepancy in the osteogenic properties may be attributed to the “site diversity” of bone tissue, but the underlying mechanisms still need further clarification. Therefore, we suggest that careful consideration should be given to the choice of animal models and cell sources in studies on osteoimmunology-mediated osseointegration *in vivo* and *in vitro*.

## Osteoimmunology in Other Systemic Disorders

**FIGURE 3:**
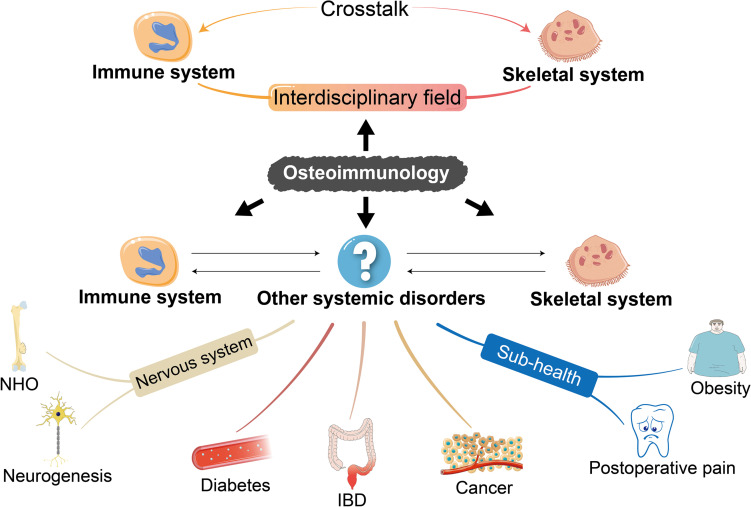
The enrichment of the concept of osteoimmunology. The understanding of some common diseases restricted to immune and skeletal systems from the perspective of osteoimmunology has been enriched in recent years. We propose that the scope of osteoimmunology should involve not only the inner mechanism of primary diseases of the skeletal and immune systems but also other systemic biological processes such as mobilopathy in diabetes, IBD, cancer, NHO, and neurogenesis as well as sub-health status like obesity and postoperative pain after tooth extraction, so as to shed light on the untapped inner mechanism and develop potential therapeutic approaches. IBD, inflammatory bowel disease; NHO, neurogenic heterotopic ossification.

Based on the clarified pathological mechanism of typical osteoimmune abnormalities, we should be aware that the co-dependent relationship of the skeletal and immune systems is also reflected in other systemic diseases, which are not fully understood. More importantly, osteoimmunology may provide a novel perspective to investigate inner mechanisms as well as to develop potential therapeutic targets for these diseases. Therefore, osteoimmunology is a theory that will not answer the whole question but will put it into a broader and multidimensional context (Figure 3).

### Mobilopathy in Diabetes

Among the systemic diseases with possible relations with osteoimmunology, “mobilopathy” described in diabetes is also reported as an underlying pathologic condition of bone niche dysfunction. The term of “mobilopathy” was first promoted by DiPersio (2011) in order to describe the failed mobilization of hematopoietic stem/progenitor cells (HSPCs) after cytokine treatment or chemotherapy, which may be associated with the female sex, agedness, etc. Besides, mobilopathy also leads to damaged endothelialization and vascular repair, and nowadays increased mortality of patients with diabetes is mainly attributed to ischemic vascular diseases (Vasam et al., 2017). Researchers have found that due to the perturbed microenvironment of bone marrow niche and interrupted dynamic regulation of CXCL12 expression, impaired mobilization of HSPCs from bone marrow to peripheral circulation is more likely to occur in individuals who are diagnosed with diabetes (Ferraro et al., 2011; Albiero et al., 2019). In the last decade, some researchers are committed to clarifying the molecular mechanism and developing novel therapeutic approach for diabetic cell mobilopathy. Fadini et al. (2013) discovered that the impaired HSPCs mobilization in diabetes was influenced by the dysregulation of DPP-4, a regulator of stromal cell-derived factor-1α (SDF-1α), in a tissue-specific manner. Additionally, the excessive expression of oncostatin M (OSM) by M1 bone marrow-derived macrophages induces CXCL12 expression, which contributes to mobilopathy in diabetes (Albiero et al., 2015). Further study indicates that role of OSM is played though OSM-p66Shc signaling, and targeting OSM-p66Shc axis may be a prospective strategy to ameliorate the mobilization of HSPCs (Albiero et al., 2019). What is more, mobilopathy can also be reflected in the impaired mobilization property of bone marrow-derived MSCs (BMSCs) in patients with type 2 diabetes, leading to inhibited bone repair and regeneration. Since it has been found that adiponectin (APN) can promote mobilization of BMSCs via the key mobilizing chemokine SDF-1, systemic infusion of APN has been demonstrated as a promising method to rescue diabetic mobilopathy and restore normal bone healing ability (Yu et al., 2015).

### Inflammatory Bowel Disease

Destruction of bone tissue is regarded as a common comorbidity of inflammatory bowel disease (IBD), which has been found to be affected by the abnormal osteoimmune microenvironment in recent years. Elevated concentrations of inflammatory factors are transported via the circulatory system and play a destructive role in regular bone metabolism, changing the protein profile in charge of bone formation and resorption and leading to osteoporosis in IBD patients (Metzger et al., 2017; van Bodegraven and Bravenboer, 2020). What is more, Ciucci et al. (2015) found that T_H_17 TNF-α^+^ cells migrated to bone marrow and promoted the recruitment of osteoclast progenitors during the process of IBD, and T cells probably mediated the crosstalk between inflamed intestine and destructed bone (Sylvester, 2017). Narayanan et al. (2018) found paralleled upregulated level of TNF-α and RANKL in gut and bone and thereby developed a novel holistic therapeutic approach dependent on the decrease of TNF-α and RANKL, suggesting the importance of paying attention to the change of immune microenvironment in bone in IBD treatment.

### Cancer

Cancer can be viewed as an important component of osteoimmunology field too. Classic osteoimmune signaling such as RANK/RANKL/OPG axis has shown its potential as the target of treatment and drug development for cancer (Croucher, 2001; Miller et al., 2014; Takayama et al., 2017). In recent years, there are researches that link osteoimmunology and cancer with other promising signaling molecules such as mTOR, estrogen-related receptor α (ERRα), and IFN signals (Irelli et al., 2019; Bouchet et al., 2020; Owen et al., 2020). Besides primary bone tumor, bone is also one of the most common metastasis sites, and the osteoimmune microenvironment of bone tissue can play a supportive role in promoting the progression of tumor in various cases. The pharmacological effects of several antitumor drugs are produced by changing the bone marrow microenvironment since cancer needs a unique metastatic niche in bone marrow to survive (Owen and Parker, 2019). For example, *E*-selectin is considered as an effective adjuvant therapeutic drug to disrupt the interaction between cancer cells and bone marrow, especially in the cancers metastasizing to the bone, stressing the importance of regulating bone marrow microenvironment in inhibiting bone metastases (Muz et al., 2021). Additionally, PTH-related peptide is highly related to bone metastasis and leads to chronic pain in patients (Shepherd et al., 2018). While it is also worth noticing that metastasized cancer cells produce PTH-related peptide to induce RANKL, thus accelerating bone resorption, in turn, bone resorption further stimulates the release of tumor growth factors, resulting in promotion of tumor growth in bone and bone metastasis (Takayanagi, 2020). In a word, it is promising to make use of osteoimmunomodulation and target the alterations of the key molecular events that will contribute to a better understanding of the exact role of osteoimmunology in cancer. Future studies are encouraged to pay attention to the crosstalk among innate immune cells, bone resident populations, and cancer cells, which may accelerate the development of therapeutic approaches.

### Nervous System

The connection between osteoimmunology and the nervous system can be reflected in neurogenic heterotopic ossification (NHO), the abnormal bone formation in periarticular muscles, which frequently develops after central nervous system (CNS) injury. Some articles have reported the promising effects of immune regulation in inhibition of heterotopic ossification (Kan et al., 2019; Sorkin et al., 2020); and current studies have established that macrophages, not neutrophils, are indispensable for NHO development (Genêt et al., 2015; Tseng et al., 2020). While the investigation of the relationship between osteoimmunology and neurogenic regulation has not been thoroughly understood, it seems that few attention is paid to neurogenic bone abnormalities, which is worth further investigation and clarification in the future.

What is more, neurogenesis in bone tissue is also a question of great clinical significance, especially in the oral implantology field. Peri-implant neurogenesis and the reestablishment of neural network is critical to the long-term application of implants for the lack of sensory functions of natural periodontal ligament between implant and bone tissue, avoiding various postoperative complications brought by excessive occlusal force. However, previous studies mainly focused on improving peri-implant osteogenesis and osseointegration between natural bone and implant, and researches on osseoperception are rare (A et al., 2018). Most importantly, suitable key targets in this progress still remain unclear. Lysophosphatidic acid (LPA), a physiologic extracellular lipid, mediate Hippo-YAP signaling to play promising regulatory roles in osteoimmunology around implants via LPA receptors (LPARs) (Zhou A. et al., 2020). As for LPAR1, a recent study showed that LPA activated macrophages via LPAR1 and affected immune response in microenvironment (Fransson et al., 2021); LPAR1 was also discovered to mediate the regulation of osteoblast function through downstream YAP signaling (Alioli et al., 2020). Therefore, we suppose that the LPA/LPAR1-YAP axis may have a prospective impact on osteoimmune-mediated osseointegration, but its specific regulatory mechanism needs further clarification. Interestingly, the latest article revealed that LPA/LPAR1 pathway initiated cilia disassembly and promoted neurogenesis by activating the transcriptional coactivators YAP/transcriptional coactivator with a PDZ-binding motif (TAZ) signaling (Hu et al., 2021), which suggests the hypothesis that the role of the LPA/LPAR1-YAP pathway in neurogenesis could be generalized to dental implantation field by establishing osseoperception in bone–implant interface in order to compensate for the missing sensory functions of periodontal ligament. Therefore, osseoperception and osteoimmunology might share the same promising signaling target and synergistically promote bone healing and favorable osseointegration, which may be achieved by interdisciplinary work such as producing a pro-neurogenesis surface, which is also friendly to osteoimmunology by surface modification of implants.

### Sub-Health Status

With the development of social economy and the improvement of medical technology, people pay more and more attention to the sub-health status and comfort diagnostic and therapeutic procedures, which raises new requirements on healthcare. For instance, obesity, a common sub-health status, has been proved to undermine both the immune and skeletal systems, and some scientists have started to investigate the inner mechanism and erase potential limitation from the point of view of osteoimmunology (Chan et al., 2012). We propose that it is of great significance to try to discover effective approaches against sub-health status, especially in association with osteoimmunomodulation.

Moreover, in order to reduce patients’ postoperative pain after tooth extraction, Wang C. W. et al. (2020) and Wang L. et al. (2020) proposed that applying endogenous proresolving mediator Maresin 1 in the extraction socket of mice could accelerate the soft tissue wound closure, promote bone fill of the socket, and preserve the alveolar bone with higher expression of CD206, the surface marker of M2 macrophage. Furthermore, researchers used an innovative method to evaluate the pain intensity of rats and proved that Maresin 1 could reduce postoperative pain (Wang C. W. et al., 2020), suggesting the study value and clinical implications to ease pain and reduce discomfort of patients from the perspective of osteoimmunology.

## Conclusion

In short, the concept of osteoimmunology can be further enriched to extend to other systemic diseases beyond the interdisciplinary field of the skeletal and immune systems. Research on the role of osteoimmunology has become the forefront topic in relevant fields since it is of great significance to discuss the pathologic biological processes of related diseases from the point of view of osteoimmunology. We suggest that researchers further clarify how to make use of the key targets in the aforementioned progresses in future studies, so as to shed light on the untapped mechanism and to develop potential therapeutic strategies for correlated diseases.

## Author Contributions

AZ designed the outline and drafted and critically revised the manuscript. BW designed the figures. HY, YT, JL, YJ, and XY contributed to drafting the manuscript. LX contributed to designing and critically revising the manuscript. All the authors contributed to the article and approved the submitted version.

## Conflict of Interest

The authors declare that the research was conducted in the absence of any commercial or financial relationships that could be construed as a potential conflict of interest.

## Publisher’s Note

All claims expressed in this article are solely those of the authors and do not necessarily represent those of their affiliated organizations, or those of the publisher, the editors and the reviewers. Any product that may be evaluated in this article, or claim that may be made by its manufacturer, is not guaranteed or endorsed by the publisher.
